# Adipocyte-Derived Paracrine Factors Regulate the In Vitro Development of Bovine Mammary Epithelial Cells

**DOI:** 10.3390/ijms241713348

**Published:** 2023-08-28

**Authors:** Żaneta Dzięgelewska-Sokołowska, Alicja Majewska, Adam Prostek, Małgorzata Gajewska

**Affiliations:** Department of Physiological Sciences, Institute of Veterinary Medicine, Warsaw University of Life Sciences (SGGW), Nowoursynowska 159b, 02-776 Warsaw, Poland; zaneta_dziegelewska-sokolowska@sggw.edu.pl (Ż.D.-S.); alicja_majewska@sggw.edu.pl (A.M.); adam_prostek@sggw.edu.pl (A.P.)

**Keywords:** bovine mammary epithelial cells, adipocytes, stroma, mammary gland development

## Abstract

The mammary gland is composed of epithelial tissue forming ducts and lobules, and the stroma, composed of adipocytes, connective tissue, and other cell types. The stromal microenvironment regulates mammary gland development by paracrine and cell–cell interactions. In the present study, primary cultures of bovine mammary epithelial cells (bMEC) and bovine adipose-derived stem cells (bASC) subjected to adipogenic differentiation were used to investigate the influence of paracrine factors secreted by preadipocytes and adipocytes on bMEC development. Four types of conditioned media (CM) were collected from undifferentiated preadipocytes (preA) and adipocytes on days: 8, 12, 14 of differentiation. Next, bMEC were cultured for 24 h in CM and cell viability, apoptosis, migratory activity, ability to form spheroids on Matrigel, and secretory activity (alpha S1-casein concentration) were evaluated. CM derived from fully differentiated adipocytes (12 d and 14 d) significantly decreased the number of apoptotic cells in bMEC population and increased the size of spheroids formed by bMEC on Matrigel. CM collected from preadipocytes significantly enhanced bMEC’s migration, and stimulated bMEC to produce alpha S1-casein, but only in the presence of prolactin. These results confirm that preadipocytes and adipocytes are important components of the stroma, providing paracrine factors that actively regulate the development of bovine mammary epithelium.

## 1. Introduction

The mammary gland is a unique exocrine gland that undergoes most development in the post-natal life of female mammals. The parenchyma of the mammary gland is made of epithelium embedded within the stroma composed of many different cell types. White adipose tissue (forming the fat pad) and connective tissue are the most abundant components of the mammary stroma. The mammary epithelial cells (MEC) can be divided into two separate lineages: luminal epithelial cells that line the ductal and alveolar lumen, forming an inner layer of cells; myoepithelial cells, also described as basal epithelial cells, that form the outer layer of ducts as well as alveoli, and are in direct contact with the stroma [1]. 

At birth, the structure of mammary epithelium is rudimentary. At this time, MEC in mice form a single network of ducts [2]. Human mammary gland contains several minor ductal networks joined at the nipple, whereas in cattle, the mammary parenchyma consists of a simple ductal network connected to a small cisternal cavity that connects to the teat cistern terminated by the teat meatus [2,3]. The prepubertal growth of the mammary epithelium is correlated with normal body growth. At the time of puberty, the growth of the mammary gland becomes positively allometric and driven by hormones of the hypothalamic-pituitary-ovarian axis. Ductal morphogenesis in the mammary gland is stimulated by estrogen and growth hormone (GH), but also prolactin and progesterone—secreted at the time of puberty due to increased levels of gonadotropin—that play an important role in acting as mitogenic factors [2]. The hormonally driven elongation and branching of the ducts takes place by intensive expansion of multicellular structures located at the tips of the ducts that invade the surrounding fat pad. In mice, these structures are termed terminal end buds (TEBs), in humans, terminal duct lobular units (TDLU), whereas in cattle, terminal ductal units (TDU) that invade the mammary fat pad with concomitant growth of the adjacent loose connective tissue [2,3]. Although the extensive ductal morphogenesis takes place in puberty, the final phases of the mammary gland development occur only during gestation, preparing the gland for future milk synthesis and secretion. At this time, the mammary gland growth is exponential and largely driven by pregnancy hormones. The mammary epithelium further expands by more extensive secondary and tertiary ductal branching, followed by development of the alveoli. Alveologenesis takes play thanks to intensively proliferating MEC that generate alveolar buds which differentiate into distinct alveoli, and become milk-secreting lobules during lactation [4]. The expansion of the mammary epithelium is accompanied by regression of the stromal interstitial adipose tissue and increased vascularization to provide a sufficient blood supply to the mammary gland at the time of lactation [4]. After lactation, weaning or cessation of milking at dry-off in dairy cattle causes milk to stagnate in the mammary epithelial cells, initiating the process of involution characterized by regression of the mammary epithelium.

The mammary gland in female mammals can undergo cycles of growth, functional differentiation, and involution several times throughout their lifespan. The development of the mammary epithelium is not only driven by endocrine actions of ovarian and pituitary hormones, but also by locally produced hormones, growth factors, and chemokines that regulate the MEC functions through paracrine mechanisms. The mammary stroma is the source of these biologically active molecules. Stromal compartment is composed mainly of adipocytes, fibroblasts, endothelial cells and immune cells which, together with extracellular matrix (ECM), form a microenvironment regulating the progress of development of the mammary epithelium. Stromal cells provide a scaffold for the parenchymal tissue, supply of nutrients, and immune defense. The paracrine, physical, and mutual signaling between MEC and underlying stromal cells regulate the proliferation, survival, polarity, differentiation, and invasive capacity of the mammary epithelium [5,6]. A well-established example of the important role of stromal paracrine actions is the local production of insulin growth factor I (IGF-I) in response to GH signaling. IGF-I is a mitogenic and antiapoptotic growth factor that supports ductal morphogenesis [7]. IGF-I is expressed in the mammary stroma during periods of ductal growth, whereas its receptor (IGF-IR) is expressed in the ductal epithelium in the pubertal mammary gland [8]. IGF-I null mice show reduced invasion of the mammary fat pad by the epithelial ductal network, but this effect can be alleviated by GH treatment [9].

Adipocytes constitute one of the most abundant cell types in the stroma of the mammary gland. In mice, adipocytes are the main cells of the mammary fat pad. In humans and cattle, the stromal adipose tissue is interlaced with predominantly present fibrous connective tissue [1,3]. In vivo models have confirmed that ductal morphogenesis is supported by adipocytes in the mammary fat pad. Lack of white adipose tissue in transgenic Z-ZIP/F1 female mice resulted in severe hypoplasia of the mammary parenchymal tissue, with only small clusters of ducts in a loose fibrous stroma [10]. On the other hand, the human mammary epithelium transplanted into the normal mammary fat pad of mice was not able to grow and form ductal network unless human stromal fibroblasts were added into the cleared fat pad [11], proving important interspecies differences.

Interactions between adipocytes and epithelial cells in the mammary gland direct the proper expansion of the mammary epithelium and control the functions of the local adipose tissue. Adipocytes isolated from the intact mammary glands of pregnant mice showed two times higher lipogenic capacity than the fat cells of nulliparous females [12]. The mammary gland adipose tissue provides a local source of fatty acids and metabolites for MEC [13]. The indirect regulation of the mammary epithelium by adipocytes is connected with their high secretory activity and local production of adipokines, such as leptin, adiponectin, chemerin, vascular endothelial growth factor (VEGF), interleukin 6 (IL-6), or tumor necrosis factor alpha (TNF-α) [13,14,15,16,17]. Deficiency in leptin signaling caused by loss of function mutation in the leptin gene causes defective mammary development. Ob/ob mice bearing this mutation have an overabundance of adipose tissue, but loss of estradiol and fertility in females [18,19]. In vitro studies with the use of Transwell co-culture model of rat mammary epithelial organoids and adipocytes demonstrated that bioactive molecules secreted by fat cells enhanced epithelial differentiation and alveolar morphogenesis [20]. Suzuki et al. [16] showed that chemerin stimulated the expression of genes associated with fatty acid synthesis, glucose uptake, insulin signaling, and casein synthesis in bovine mammary epithelial cells.

The majority of studies on the role of stromal cells and local microenvironment in development of the mammary epithelium have been based on rodent models of co-transplantation or/and genetic modifications. These models cannot be used to infer results for other species, as there are differences in the morphology and physiology of the mammary gland [19,21]. Additionally, studies using cell cultures have given insight into how adipokines and direct interactions between adipocytes and epithelial cells affect the growth, survival, polarity, and differentiation of MEC. However, the cells used in these studies are mostly from mice or humans. Information about the role of stromal cells in regulation of bovine mammary epithelium development is still scarce, although the bovine mammary gland is one of the most exploited by milk production that constitutes an important sector of the worldwide agricultural production [22]. Therefore, the aim of the present study was to determine the influence of paracrine factors secreted by preadipocytes and adipocytes on viability, proliferative activity, and secretory activity of bovine mammary epithelial cells (bMEC) using in vitro models of primary bovine cell cultures.

## 2. Results

### 2.1. Morphological and Molecular Features of Bovine Mammary Epithelial Cells Isolated from the Mammary Gland Tissue

**Figure 1:**
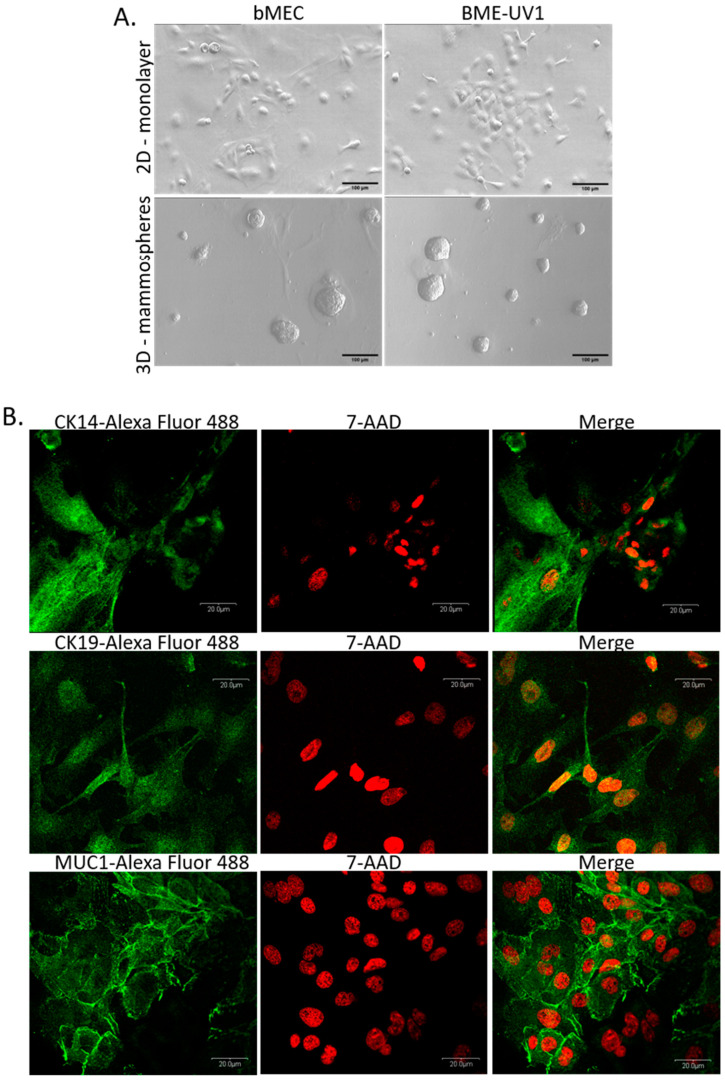
Morphology of bovine mammary epithelial cells. (**A**) Monolayer and 3D cultures of primary bovine mammary epithelial cells (bMEC)—passage 3, and control cell line: BME-UV1 of immortalized bovine mammary epithelial cells; in 3D cultures, cells were grown on Matrigel. Phase-contrast micrographs were taken at 200× magnification in the case of 2D culture, and at 100× magnification in the case of 3D culture. Scale bars represent 100 µm (**B**) Markers of bovine mammary epithelial cells—panels of confocal micrographs presenting immunofluorescence staining of bMEC with antibodies against cytokeratin 14 (CK14)—marker of myoepithelial cells (upper panel), cytokeratin 19 (CK19)—marker of luminal mammary epithelial cells (middle panel), and MUC1—marker of luminal mammary epithelial cells (lower panel). Secondary antibodies were conjugated with Alexa Fluor 488 dye (green fluorescence), nuclei were counterstained with 7-aminoactinomycin D (7-AAD, red fluorescence). Images were taken at 600× magnification and are representative for three independent experiments.

The mammary epithelial cells used in this study were isolated from the bovine mammary gland. Bovine mammary epithelial cells (bMEC) showed a typical cobblestone morphology when grown on standard plastic surface and closely resembled the morphology of the established bovine mammary epithelial cell line: BME-UV1 (Figure 1A). Furthermore, the primary culture of bMEC formed three-dimensional (3D) spherical organoids (mammospheres) when cultured on reconstituted basement membrane (Matrigel), similarly to BME-UV1 cells (Figure 1A). Immunofluorescence staining of bMEC confirmed the expression of mammary epithelial cell markers: cytokeratin 14, cytokeratin 19, and MUC1 (Figure 1B). Reverse transcription-quantitative PCR (RT-qPCR) also showed that bMEC express genes encoding cytokeratins: KRT5, KRT14, KRT18, and KRT19 (Supplementary Figure S1). Thus, after confirming the characteristic features of the primary bMEC, these cells were used in further experiments.

### 2.2. Morphological and Biochemical Features of Preadipocytes Isolated from the Bovine Perirenal Fat Tissue and Characteristics of Bovine Adipocytes

**Figure 2:**
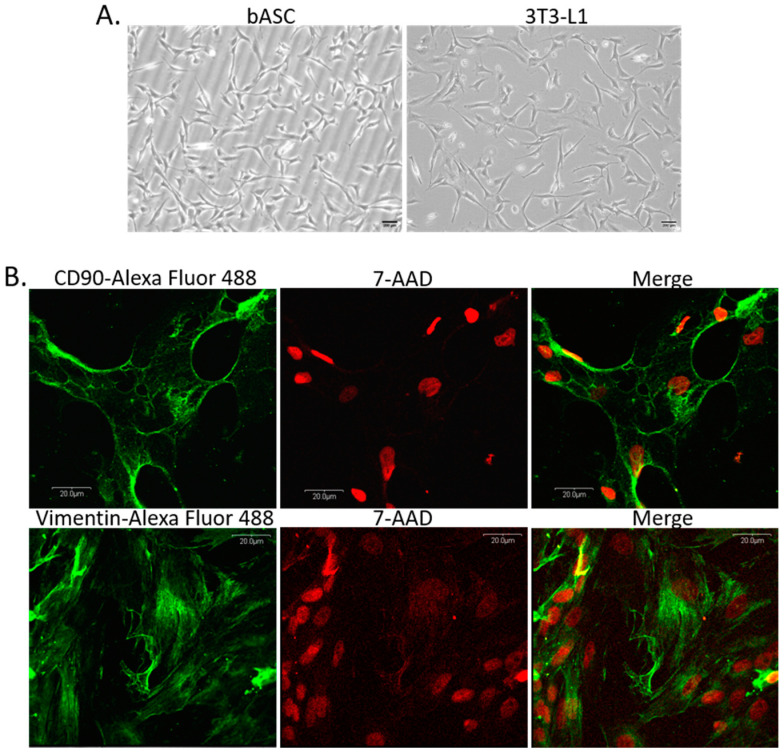
(**A**) Morphology of monolayer cultures of bovine adipose-derived stem cells (bASC)—passage 3, and 3T3-L1 murine preadipocyte cell line used as a reference cell line for adipogenic differentiation. Phase-contrast micrographs were taken at 100× magnification, scale bars represent 200 µm. (**B**) Panels of confocal micrographs presenting immunofluorescence staining of bASC with antibodies against CD90 (upper panel) and vimentin (lower panel). Secondary antibodies were conjugated with Alexa Fluor 488 dye (green fluorescence), nuclei were counterstained with 7-AAD (red fluorescence). Images were taken at 600× magnification and are representative for three independent experiments.

**Figure 3:**
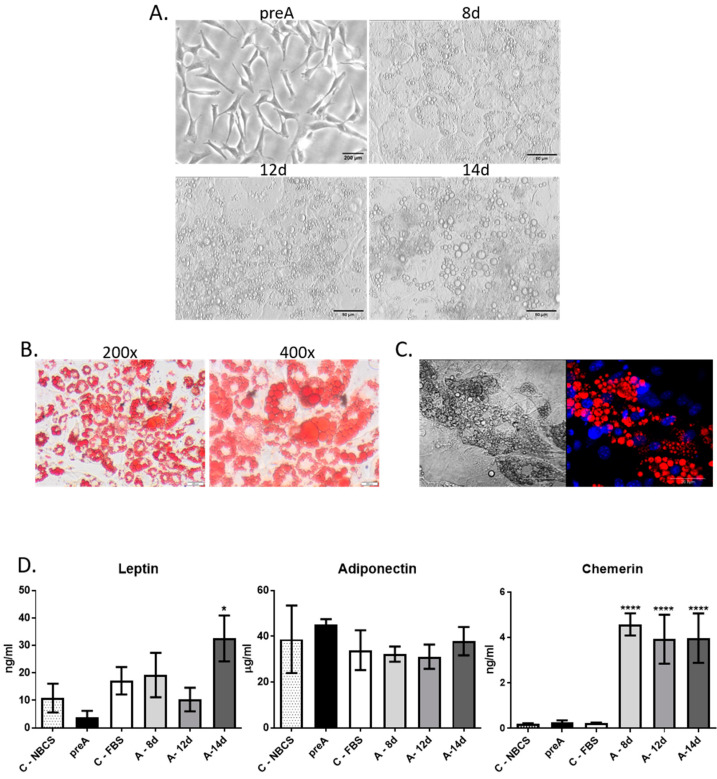
Adipogenic differentiation of bASC. (**A**) Morphology of monolayer cultures of undifferentiated bASC described as preadipocytes (preA) and bASC subjected to in vitro adipogenic differentiation (as described in the text) for 8, 12, or 14 days. Phase-contrast micrographs were taken at 200× magnification. Scale bar in image presenting preadipocytes represents 200 µm, whereas in images presenting adipocytes on days 8, 12 and 14 of differentiation the scale bars represent 50 µm (**B**) Phase-contrast images of bovine adipocytes on day 12 of differentiation stained with Oil Red O to detect neutral lipids—images were taken at 200× and 400× magnification. Scale bars represent 200 µm (**C**) A representative confocal image of LipidTOX^TM^-stained lipid vacuoles (red fluorescence) in bovine adipocytes on day 12 of bASC differentiation (right micrograph) with nuclei counterstained with Hoechst 33342 (blue fluorescence); left micrograph presents the same cells in Nomarski interference contrast. Images were taken at 600× magnification and are representative for three independent experiments. Scale bars represent 20 µm (**D**) Concentration of selected adipokines: leptin, adiponectin, chemerin in conditioned media (CM) derived from 24 h culture of bovine preadipocytes (preA) and adipocytes on different days of differentiation (8 d, 12 d, 14 d). Fresh culture media supplemented with 10% NBCS or 10% FBS were used as controls, to exclude the levels of adipokines in sera present in the media. Results are presented as means ± standard deviation of three separate experiments. Values that differed significantly from both types of control media are marked as * (*p* < 0.05), **** (*p* < 0.0001).

Cells isolated from the perirenal fat tissue showed a spindle-shaped morphology when cultured on plastic surface, which is typical for cells of mesenchymal origin (Figure 2A). Cells described by us as bovine adipose-derived stem cells (bASC) had similar morphology to 3T3-L1 murine cell line of preadipocytes that have a fibroblast-like morphology, and are able to undergo in vitro adipogenic differentiation (Figure 2A). Immunofluorescence staining of bASC with antibodies against CD90 and vimentin indicated that these cells show characteristics of the mesenchymal stem cells (Figure 2B). Most importantly, bASC were able to differentiate into adipocytes when cultured in the medium supplemented with factors stimulating adipogenesis (isobutylmethylxanthine, dexamethasone, insulin-transferrin-selenium, and rosiglitazone) (Figure 3). Cells before adipogenic stimulation were referred to as preadipocytes, showing typical fibroblast-like morphology. During adipogenic differentiation, bASC became rounder, showing signs of lipid accumulation in the form of fat vacuoles (Figure 3A). Cells from day 8 (8 d) of differentiation were described as poorly differentiated adipocytes (young adipocytes with small fat vacuoles). On day 12 (12 d) of differentiation, cells showed multiple, regular, large fat vacuoles. On day 14 (14 d), mature adipocytes showed one dominant fat vacuole, although other smaller vacuoles were still visible (Figure 3A). Oil red O staining as well as fluorescence staining with LipidTOX^TM^ confirmed accumulation of lipids in differentiating bovine adipocytes (Figure 3B,C).

To assess the secretory potential of bovine preadipocytes (preA) and adipocytes at different stages of differentiation, we analyzed the presence of three adipokines: leptin, adiponectin, and chemerin (also known as retinoic acid receptor responder protein 2—RARRES2) in the conditioned media (CM) collected at different stages of bovine adipocytes differentiation (preA, 8 d, 12 d, and 14 d). Adipokines were chosen based on their documented role in regulation of mammary epithelium development [15,16,23,24]. Analyses also included fresh culture media supplemented with 10% new born calf serum (NBCS) or 10% fetal bovine serum (FBS) in order to estimate the concentrations of adipokines in sera used in the cell culture. Immunoenzymatic tests showed the highest concentration of leptin in CM collected from mature adipocytes with large lipid accumulation (14 d) (Figure 3D). Surprisingly, no significant changes were observed in adiponectin concentration among tested media (Figure 3D). In the case of chemerin, the concentration was significantly higher in adipocytes, compared to control conditions and to CM collected from preadipocytes (Figure 3D). Taken together, the results confirmed that CM derived from preadipocytes’ and adipocytes’ culture were enriched with biologically active compounds secreted by these cells. Therefore, in the next part of this research, the CM collected after 24 h culture of bovine preadipocytes and adipocytes were used to investigate the paracrine effect of these cells on bovine mammary epithelial cells.

### 2.3. Effect of Paracrine Factors Secreted by Bovine Preadipocytes and Adipocytes on Growth and Viability of bMEC

**Figure 4:**
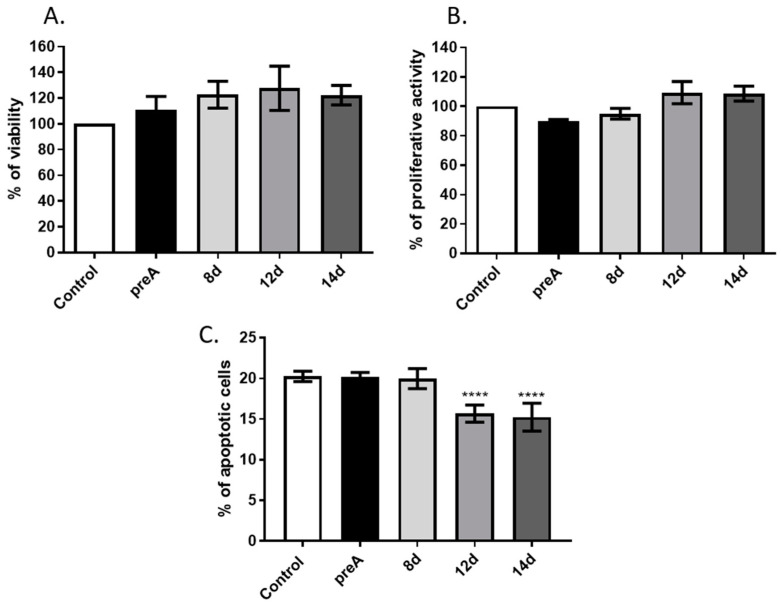
Effect of conditioned media (CM) derived from 24 h culture of bovine preadipocytes (preA) and adipocytes on different days of differentiation (8 d, 12 d, 14 d) on basic viability parameters of bMEC. Graphs present: (**A**) Cell viability measured by MTT assay, (**B**) proliferative activity measured by CyQUANT^®^ Proliferation Assay, (**C**) percentage of apoptotic cells (Annexin V^pos^/PI^pos^) in bMEC population measured by Annexin V assay. In graphs A and B, control values were designated as 100%. Results are presented as means ± standard deviation of three independent experiments (in the case of Annexin V assay, the experiment was performed four times). Values that differed significantly from control are marked as **** (*p* < 0.0001).

Stromal cells of the mammary gland actively participate in regulation of the mammary epithelium development via synthesis and secretion of active biomolecules (e.g., hormones, growth factors, chemokines), as well as through direct cell–cell interactions. In the present study, we investigated the effect of paracrine actions of undifferentiated preadipocytes as well as adipocytes on bMEC growth and viability. In the first step, we analyzed the influence of conditioned media collected from preadipocytes (preA) and adipocytes at different stages of differentiation (8 d, 12 d, 14 d) on viability, proliferative activity, and apoptotic cell death of bMEC. Results of MTT assay did not show any significant differences in viability of bMEC grown in standard growth medium or in CM collected from preadipocytes (preA) and adipocytes (8 d, 12 d, 14 d) (Figure 4A). To test the proliferative activity of bMEC, the CyQUANT^®^ Proliferation Assay was used, which is based on the measurement of cellular DNA content using a dye exhibiting fluorescence when bound to DNA. Proliferative activity of bMEC did not change significantly after treatment with the experimental conditioned media in comparison to control (Figure 4B). Annexin V assay was used to determine the percentage of apoptotic cells in cultured bMEC population (Figure 4C). The results showed significantly decreased number of apoptotic cells (Annexin V^pos^/PI^pos^) when bMEC were cultured in CM collected from mature adipocytes (12 d and 14 d), indicating an anti-apoptotic effect of paracrine factors synthetized by adipocytes (Figure 4C).

### 2.4. Effect of Paracrine Factors Secreted by Bovine Preadipocytes and Adipocytes on bMEC Motility

**Figure 5:**
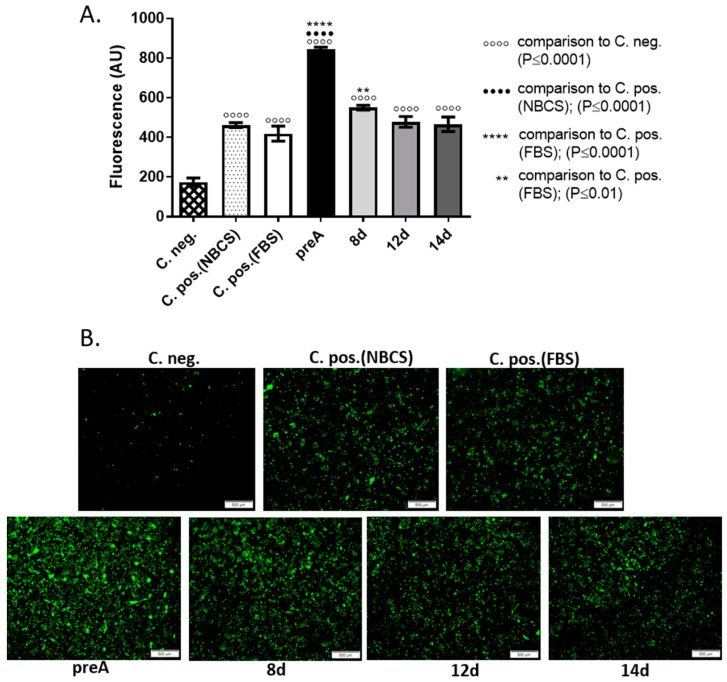
Effect of conditioned media (CM) derived from 24 h culture of bovine preadipocytes (preA) and adipocytes on different days of differentiation (8 d, 12 d, 14 d) on bMEC migration. (**A**) Graph presenting the fluorescence intensity of bMEC that migrated through the insert’s membrane and were dyed with calcein AM. Results are presented as means ± standard deviation of three separate experiments. (**B**) Representative micrographs showing bovine mammary epithelial cells that passed through the porous membrane. Upper panel presents images of cells in control conditions: C.neg—control medium without chemoattractant (serum), C. pos. (NBCS)—positive control (culture medium with 10% NBCS), C. pos. (FBS)—positive control (culture medium with 10% FBS); lower panel presents bMEC cultured in experimental CM. All images are representative for three separate experiments. Scale bars represent 500 µm.

Cell migration is one of the important properties of epithelial cells during morphogenesis of the mammary gland [25,26,27]. With the onset of puberty, a combination of systemic and paracrine factors induce terminal end buds to reappear at the ductal tips which is accompanied by a significant increase in the growth rate of the epithelial tissue. Elongation and branching of the ducts, regulated by proliferation and migration of cells, rely on both endocrine and local growth regulatory signals, ECM remodeling, and contact with the stroma [2]. Therefore, we investigated how paracrine factors synthesized and secreted by bovine preadipocytes and adipocytes at different stages of differentiation affect the ability of bMEC to migrate through a porous membrane in an insert culture system. The conditioned media collected from each type of cell culture (preA, 8 d, 12 d, and 14 d) served as the potential source of chemoattractants. The bovine mammary epithelial cells crossed light-tight polyethylene terephthalate membrane, in the direction of selected CM. Cells that migrated through the membrane, were dyed with calcein AM and detected by a bottom-reading fluorescence plate reader. In addition, series of images were taken using an inverted fluorescence microscope (Figure 5). Since the presence of serum in the culture media can be a potent chemoattractant inducing cell migration, we used three types of controls in this experiment: negative control—culture medium without serum; positive control—culture medium with 10% NBCS (medium used for preadipocytes culture); positive control—culture medium with 10% FBS (medium used for culture of adipocytes). Analysis of fluorescence intensity revealed a highly significant increase in migration of bMEC in the direction of CM from preadipocytes when compared with all other controls and tested conditions (Figure 5A). A significant increase in bMEC migration was also observed when cells were treated with CM from young adipocytes (8 d) in comparison to the positive control with FBS; however, the effect was less pronounced than in the case of CM from preA culture (Figure 5A). The micrographs taken after measurement of the fluorescent intensity by a plate reader confirmed the overall trend (Figure 5B), showing the largest number of bMEC (stained green with calcein AM) that had passed the membrane towards the preA conditioned medium.

### 2.5. The Role of Preadipocytes and Adipocytes in Regulation of Development of Mammospheres by bMEC Cultured on Matrigel

**Figure 6:**
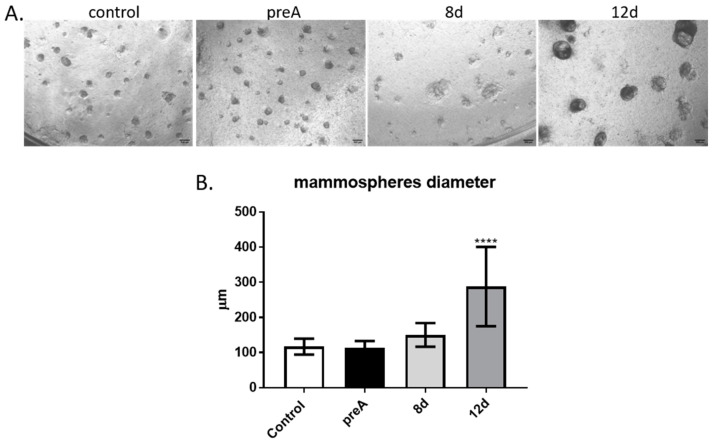
Effect of paracrine actions of bovine preadipocytes (preA) and adipocytes on different days of differentiation (8 d, 12 d) on bMEC’s ability to form mammospheres when cultured on Matrigel. (**A**) bMEC cultured for 6d on inserts coated with Matrigel, grown above wells in which bASCs were cultured at different stages of adipogenic differentiation: prior differentiation (preA), on day 8 or day 12 of differentiation (8 d, 12 d, respectively). Control cells were grown in standard growth medium for bMEC. Images are representative for three separate experiments (40× magnification was used). Scale bars represent 500 µm. (**B**) Diameters of mammospheres formed by bMEC cultured for 6 days on inserts coated with Matrigel, grown above wells with cultured bovine preadipocytes or adipocytes at different stages of differentiation. Results are presented as means ± standard deviation of three separate experiments, in which diameters of at least 10 mammospheres per each experimental condition were measured. Values that differed significantly from control are marked as **** (*p* < 0.0001).

In order to determine the effect of paracrine factors secreted by the bovine adipocytes at different stages of development on the ability of bMEC to form acini-like spheroids (mammospheres), we performed another experiment with the use of insert co-culture system. In this culture model, bMEC were grown on Matrigel-covered inserts, whereas preadipocytes or adipocytes were cultured in the lower chambers of 12-well companion plates. Inserts with bMEC seeded on Matrigel were placed above the wells of the companion plates containing preadipocytes (preA) or adipocytes at different stages of adipogenic differentiation (8 d, 12 d). Micrographs of mammospheres formed by bMEC were taken on day 6 of bMEC’s culture on the Matrigel-covered inserts (Figure 6A). In control conditions, the culture medium without bASC was placed in the lower chamber. Diameters of the mammospheres were measured in each culture condition (Figure 6B). The results showed that bMEC seeded on Matrigel and placed above wells containing differentiated adipocytes (12 d) formed significantly larger mammospheres within 6 days of culture when compared with other experimental and control conditions (Figure 6).

### 2.6. Effect of Paracrine Factors Secreted by Bovine Preadipocytes and Adipocytes on Secretory Activity of bMEC

**Figure 7:**
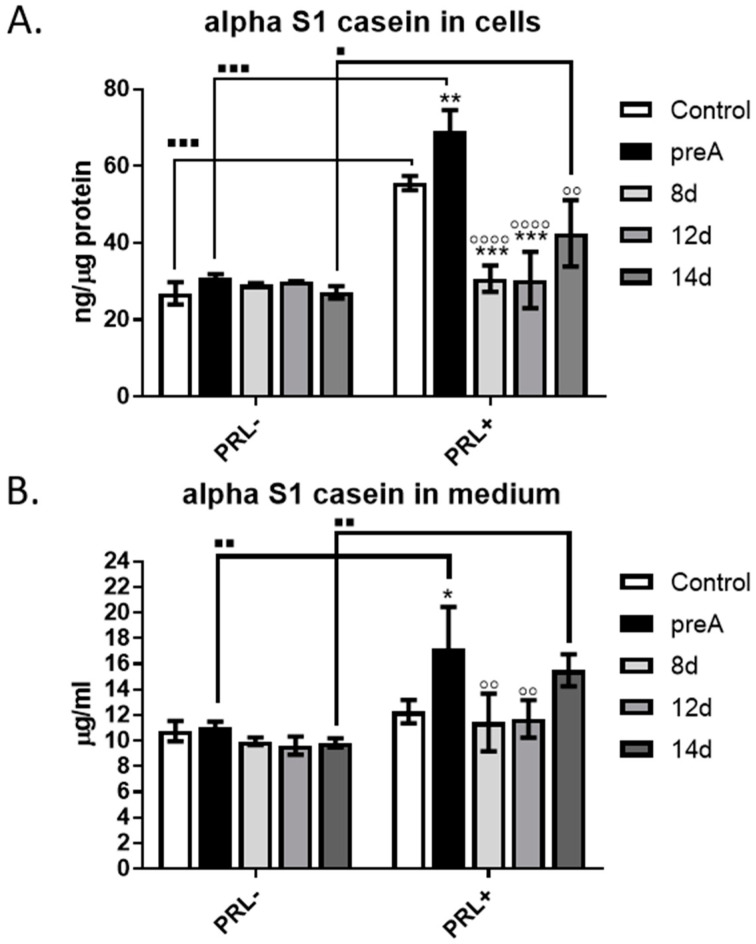
Effect of conditioned media (CM) derived from 24 h culture of bovine preadipocytes (preA) and adipocytes on different days of differentiation (8 d, 12 d, 14 d) on synthesis and secretion of alpha S1-casein by bMEC. Graphs present concentration of alpha S1-casein in: (**A**) cells after 24 h of treatment; (**B**) culture medium collected after 24 h of treatment. In this experiment, prolactin (PRL, 3 µg/mL) was used as an additional prolactogenic factor. Results are presented as means ± standard deviation of three independent experiments. Values that differed significantly within one type of treatment (PRL− or PRL+) in comparison to control are marked as * (*p* < 0.05), ** (*p* < 0.01), or *** (*p* < 0.001). Values that differed significantly from preA in cells treated with prolactin (PRL+) are marked as ◦◦ (*p* < 0.01), or ◦◦◦◦ (*p* < 0.0001). Values that differed significantly between two types of treatments (PRL− and PRL+) are marked as ∎ (*p* < 0.05), ∎∎ (*p* < 0.01), or ∎∎∎ (*p* < 0.001).

Functional differentiation of the mammary epithelial cells is reflected by their ability to synthesize and secrete milk components. Thus, we investigated the effect of paracrine factors produced by bovine adipocytes at different stages of differentiation (preA, 8 d, 12 d, 14 d) on the secretory activity of bMEC. Bovine milk contains more than twenty five different proteins, among which caseins are the most abundant. In the present study, alpha S1-casein, one of the major milk proteins produced in the bovine mammary gland [28], was chosen as a marker of the functional differentiation. Concentration of alpha S1-casein was measured in the lysates from bMEC cultured for 24 h in the conditioned media collected from preadipocytes and adipocytes at different stages of differentiation (preA, 8 d, 12 d, 14 d), as well as in the media collected after the experimental culture of bMEC (Figure 7). The effect of paracrine factors synthesized and secreted by adipocytes was compared with the effect of lactogenic hormone—prolactin (PRL, 3 µg/mL). Thus, two types of CM were used: supplemented (PRL+) or not supplemented (PRL−) with prolactin. Results demonstrated that without addition of PRL, there were no significant differences in the concentration of alpha S1-casein in the cells or in the media collected after 24 h culture of bMEC compared to control (Figure 7A,B, respectively). Administration of PRL significantly stimulated the synthesis and secretion of alpha S1-casein, in the control conditions as well as in bMEC cultured in CM collected from preadipocytes (preA + PRL) and mature adipocytes (14 d + PRL) (Figure 7). When bMEC were cultured in CM collected from adipocytes on day 8 and 12 of differentiation (8 d + PRL, 12 d + PRL), a significantly lower concentration of alpha S1-casein was observed in cells and medium compared to control + PRL and preA + PRL conditions (Figure 7). The highest concentration of the milk protein was noted in cells and medium when bMEC were cultured in the CM from preadipocytes supplemented with PRL (preA + PRL). This may suggest a synergistic effect of PRL and the paracrine factors present in preA-derived conditioned medium that stimulated the secretion of alpha S1-casein above the levels found in bMEC treated only with PRL in the control conditions (Figure 7).

## 3. Discussion

The importance of mammary gland stroma in the development and function of epithelial cells is becoming more recognized in recent years. Most research on this topic uses rodent or human experimental models, leaving the role of stromal cells in bovine mammary epithelium development and function largely unexplored. Although the basic structure of the mammary gland is similar in most mammals, specific morphological features vary substantially. In rodents, the mammary gland stroma, called fat pad, is mainly composed of adipocytes, whereas in humans and cattle, the adipocytic compartment is relatively less represented since stroma contains more fibrous connective tissue [21]. Nevertheless, based on the studies using different transgenic mouse models or tissue samples isolated from humans with lipodystrophy, it has been established that mammary gland adipose tissue is required for proper development at puberty and remodeling during the lactation cycle. Both the secretory activity of adipocytes and direct cell–cell interactions between epithelial cells and fat cells of the stroma are the key factors regulating the mammary gland development [19,21]. To date, very few studies have been conducted with the mammary gland parenchymal and stromal cells of farm animals. It is important to understand the development of the mammary epithelium and tissue plasticity during lactation cycles in dairy cattle, to regulate more efficiently the milk yield and animal welfare. In our study, we examined how two types of stromal cells, preadipocytes (with fibroblast-like morphology) and adipocytes at different stages of differentiation, affect the development of the bovine mammary epithelial cells. We used in vitro model of primary cell culture. Thus, we isolated bMEC and bASC from bovine tissues (mammary tissue from the udders and adipose tissue from the adipose capsule of bovine kidneys, respectively). Prior to the main experiments, the primary cells had been characterized based on chosen molecular markers. bMEC showed morphological features comparable to the established cell line of bovine mammary epithelial cells: BME-UV1 [29,30]. Isolated epithelial cells were able to form 3D spherical organoids (mammospheres) when cultured on Matrigel, similarly to BME-UV1 cells [31]. bMEC also expressed MUC1—a major mucin glycoprotein expressed on the surface of mammary epithelial cells [32,33], cytokeratin 19—one of the main cytoskeleton proteins of luminal epithelial cells, as well as cytokeratin 14—characteristic for myoepithelial cells and basal mammary epithelial cells [30,34]. Furthermore, analysis of cytokeratin gene expression in bMEC (supplementary Figure S1) revealed the expression of four genes: *KRT5, KRT14, KRT18*, and *KRT19*, confirming that the population of primary bMEC was heterogenous and comprised epithelial cells of luminal origin (confirmed by the expression of *KRT18* and *KRT19*) and basal origin (myoepithelial cells expressing *KRT5* and *KRT14*) [34,35,36]. Bovine adipose-derived stem cells expressed CD90 (a surface glycoprotein) and vimentin (a cytoskeletal class III intermediate filament). Both of these proteins are expressed by cells with fibroblastic morphology, and are used as markers of the mesenchymal stem cells [37,38,39]. The stemness of bASC was confirmed by their ability to differentiate into adipocytes showing characteristic fat vacuoles and the ability to secrete adipokines.

Based on available literature, we chose to analyze the concentration of three selected adipokines: leptin, adiponectin and chemerin, in the conditioned media collected from bASC at different stages of adipogenic differentiation (preA, 8 d, 12 d, 14 d). Leptin was chosen since studies had demonstrated that mice lacking the expression of leptin (ob/ob) or its receptor (db/db) are infertile and have severely underdeveloped mammary tissue [18,40]. The expression of leptin transcript was also described in the mammary adipocytes of lactating, nonpregnant cows as well as in the dry period [41]. Adiponectin belongs to one of the most abundant adipokines in circulation, and its serum concentration is inversely connected with the mass of body fat [42]. This protein hormone is also locally expressed in the parenchymal and stromal compartment of the bovine mammary gland during lactation [43], suggesting the role of adiponectin in the paracrine regulation of MEC differentiation. Jeong et al. [24] showed an increase in proliferative activity and cell cycle progression in MAC-T bovine mammary epithelial cells treated with adiponectin. The last adipokine that was analyzed in the conditioned media was chemerin—a chemokine expressed and secreted by white adipose tissue of several species, including mice, humans, and cattle [44,45,46,47]. Elis et al. [48] reported a greater chemerin expression in the adipose tissue of post-partum dairy cows than in pregnant cows, whereas Suzuki et al. [16] demonstrated the expression of chemerin receptors in the bovine mammary epithelial cells. For the purpose of this study, the selected adipokines were analyzed in CM collected after 24 h culture of confluent preadipocytes or adipocytes at different stages of differentiation. In addition, two types of control samples were used in the immunoenzymatic assays: the culture medium supplemented with 10% NBCS or 10% FBS (used in preadipocytes or adipocytes culture, respectively). The control media were unmetabolized and contained all components of the DMEM/F12 medium as well as compounds present in the specific sera used as supplements. We did not use serum-free media in our experiments to maintain the recommended cell culture conditions for preadipocytes and adipocytes. Serum (NBCS or FBS) in the culture media is essential for cell attachment and survival as it provides hormones, growth factors, extracellular matrix components, carrier proteins, and trace elements [49,50]. Nevertheless, the presence of serum in the media can also be an additional source of adipokines and chemokines, thus we used the fresh/unmetabolized media (with 10% NBSC or 10% FBS) as controls, to determine whether the concentration of selected adipokines resulted mainly from the secretory activity of preadipocytes and adipocytes, or originated from the serum. In our study, leptin and chemerin were secreted specifically by differentiated bovine adipocytes. The concentration of leptin in CM derived from the 24 h culture of preadipocytes was lower than in the control medium for these cells (with 10% NBCS), indicating that preadipocytes were not capable of synthesizing and secreting leptin, and the detected hormone originated from the serum. The concentration below the level detected in the control samples suggests that leptin originating from NBCS was partially absorbed by preadipocytes within the 24 h culture, and the secretion of this adipokine did not take place. Surprisingly, the concentration of adiponectin did not differ significantly among all investigated conditions, and was similar to the control media used in this experiment. Adiponectin is considered as being produced solely by mature adipocytes [51]; however, some researchers demonstrated that this hormone may be also produced to some extent by other cell types, e.g., osteoblasts, myocytes, cardiomyocytes, or in bone marrow [52]. Yamazaki et al. [53] detected this adipokine in the culture media derived from 3T3-L1 murine preadipocytes and human subcutaneous preadipocytes. Results of our study did not univocally confirm that adiponectin was actively produced by bovine adipocytes. Nevertheless, the results of immunoenzymatic tests confirmed that CM derived from in vitro cultures of bovine preadipocytes and adipocytes contained active compounds (among others: leptin and chemerin) that could potentially regulate growth and differentiation of other cells. Therefore, in the next step of our research, we used conditioned media derived from preadipocytes and adipocytes to investigate the paracrine interactions between bMEC and adipocytes at different stages of differentiation.

CM collected from preadipocytes (preA) did not affect bMEC viability (analyzed by MTT test) or proliferative activity. A slight increase in both parameters could be noted when bovine mammary epithelial cells were treated with CM from differentiated adipocytes; however, this tendency was not statistically significant. Similar observations were described in the case of murine NMuMG mammary epithelial cells grown in CM derived from 3T3-L1 cells at various degrees of differentiation: preadipocytes, poorly differentiated adipocytes, and mature adipocytes [54]. After 24 h of incubation, murine MEC showed higher proliferative activity when cultured in CM from mature adipocytes, whereas 48 h of incubation significantly elevated proliferative activity of NMuMG cells cultured in CM derived from mature adipocytes [54].

Although we did not observe statistically significant changes in the proliferative activity of bMEC, our study demonstrated a significant decrease in the number of apoptotic cells (Annexin V^pos^/PI^pos^) when bMEC were cultured for 24 h in CM collected from mature adipocytes (12 d and 14 d of differentiation). This indicates an anti-apoptotic, and thus cytoprotective effect of the paracrine factors synthetized by mature adipocytes. Based on the results obtained, it is not possible to state specifically which active compounds, found in the adipocytes-derived CM, contributed to the lower number of apoptotic cells in bMEC population. Leptin, found in the highest concentration in CM collected from adipocytes on day 14 of culture, may be one of the adipokines exerting anti-apoptotic activity in bMEC. Thorn et al. [40] observed that lack of signals induced by leptin, caused by mutation at tyrosine 1138 of the Ob-Rb receptor, resulted in a complete arrest of the mammary ductal growth in mice. In our study, adipocytes also secreted chemerin at concentrations >6 ng/mL. Hu et al. [18] previously observed apoptosis induction in bMEC after 24 h treatment with chemerin. However, this research group used chemerin in concentration of 100 ng/mL, which is over 15-fold higher than the level detected in CM analyzed in our study. The anti-apoptotic effect of paracrine factors produced by mature adipocytes may also be a resultant of multiple signals acting in an agonistic manner, and this topic should be further investigated in the future.

Our research also showed that treatment with CM collected from preadipocytes significantly increased bMEC motility, suggesting the presence of strong chemotactic factors in the CM. Epithelial cell migration is crucial for the mammary gland development, especially during elongation and branching of the ducts [25,26,27]. Fibroblasts present in the mammary stroma are important regulators of branching morphogenesis [55]. These cells, as well as preadipocytes, synthesize and secrete fibroblast growth factors (FGFs) that induce signaling pathways essential for vertebrate organogenesis, including mammary gland development [56]. FGF family comprises eighteen secreted proteins, that interact with FGF receptors (FGFRs) with tyrosine kinase activity. Studies have shown that FGF2, FGF7, FGF9, and FGF10 enhance murine mammary epithelial cells migration and expansion, crucial for branching morphogenesis [56,57]. When FGF2 was used in an in vitro study, murine mammary organoids grown on Matrigel formed more branched structures at a higher rate [56]. Although we did not examine the presence of FGFs in the preA-derived CM, it is highly probable that these growth factors contributed to the observed marked increase in bMEC motility. Further research is necessary to identify the biologically active compounds in CM collected from preadipocytes culture and their specific role in regulating the development of bovine mammary gland epithelium.

During pregnancy and lactation, the mammary epithelium expands undergoing intensive elongation and branching of the ducts as well as forming the lobuloalveolar structures producing milk. Alveologenesis is driven by multiple factors, including endocrine regulation by hormones of the hypothalamic-pituitary-ovarian axis, as well as paracrine molecules (growth hormones, cytokines, adipokines) locally produced by stromal cells [14,19,58]. We used an insert co-culture system to investigate the paracrine effect of preadipocytes and adipocytes on the ability of bMEC to form mammospheres. In our experiments, bMEC were seeded on inserts covered with Matrigel, and placed above wells containing preadipocytes or adipocytes at different stages of differentiation (8 d—young adipocytes, 12 d—well-differentiated adipocytes). Results clearly demonstrated that well-differentiated adipocytes (12 d) significantly enhanced the growth of 3D spheroids formed by bMEC on Matrigel, supporting the hypothesis of the important function of adipocytes in proper functional development of the mammary epithelium. The knowledge about the role of adipokines in regulation of alveologenesis at the time of pregnancy is still scarce. Brenot et al. [19] demonstrated that direct contact between the mammary epithelial cells with adipocytes, as well as the presence of leptin and estradiol, are required for the mammary gland development. In lipodystrophic (LD) mice, the underdeveloped phenotype of the mammary gland resulted from the lack of adipocytes in the glandular microenvironment, not due to an inherent defect in the mammary epithelium [19]. This had been experimentally proven by transplantation of MEC isolated from LD mice into a wild-type (WT) host, and observation that the mammary epithelium was able to grow and expand in the WT fat pad [19]. Ob/ob mice that have a loss of function mutation in the leptin gene are characterized by an overabundance of adipose tissue. Lack of leptin in these animals causes a secondary loss of estradiol, resulting in inhibition of development of the mammary epithelium [18]. There are no corresponding experimental models that could be used to confirm these observations in cattle. However, our results encourage more research on specific adipokines supporting mammary epithelium development in cows during pregnancy and lactation.

In the last step of our research, we evaluated the effect of CM derived from preadipocytes (preA) and adipocytes at various degrees of differentiation (8 d, 12 d, 14 d) on the ability of bMEC to synthesize and secrete milk proteins. We analyzed the concentration alpha S1-casein in both bMEC and the culture medium derived from bMEC’s 24 h culture. The results did not show any significant effect of the tested CM when the lactogenic hormone was not added. However, when the conditioned media were additionally supplemented with PRL, a significant increase in alpha S1-casein synthesis and secretion was noted in bMEC cultured in preA-derived CM (preA + PRL), in CM collected from fully differentiated adipocytes (14 d + PRL), as well as in the control conditions (Control + PRL). The highest concentration of alpha S1-casein in cells and in the media collected after 24 h culture of bMEC was observed when the mammary epithelial cells were cultured in preA-derived CM supplemented with PRL. This may suggest a synergistic effect between PRL and the paracrine factors present in the conditioned media derived from preadipocytes. The full content of preadipocyte-derived CM was not analyzed for the purpose of this study. However, some stroma-derived growth factors identified by other research groups may act as molecules involved in the observed increase in alpha S1-casein concentration in bMEC culture medium. IGF-I and members of the FGF family are among possible candidates, as these growth factors are known to be locally synthesized by the mammary gland stroma [59]. IGF-I stimulates DNA binding activity of one of the key transcription regulators of casein genes: STAT5 (signal transducer and activator of transcription 5), which belongs to the Janus kinase—signal transducer and activator of transcription pathway (JAK2/STAT5 pathway) stimulated by prolactin [60]. In cattle, IGF-I is synthesized by stromal tissue cells surrounding the mammary ducts [59]. In addition, several members of the FGF family are important stroma-derived mitogens involved in ruminant mammary gland development [59]. Jeong et al. [61] used an in vitro model of MAC-T bovine mammary epithelial cells to study the signaling pathways induced by FGF-2. The authors showed that FGF-2 stimulated bMEC proliferation by activation of signaling pathways mediated by protein kinase B (PKB), extracellular signal–regulated kinases 1 and 2 (ERK1/2), and Jun N-terminal kinase (JNK), and suggested that this growth factor may be important for increasing persistency of milk production [61]. Although it cannot be clearly stated which factors present in the CM collected from preadipocytes were responsible for enhanced production of alpha S1-casein above the level induced by PRL, our results indicate that the paracrine activity of the stromal cells is also involved in regulation of lactogenesis in the bovine mammary gland. Further studies will be conducted by our research group to investigate the expression of IGF-I and FGF in bovine preadipocytes.

When analyzing the concentration of alpha S1-casein in bMEC and corresponding culture media, we also observed a significantly lower concentration of the milk protein in bMEC grown in CM collected from bovine adipocytes at earlier stages of differentiation (8 d + PRL, 12 d + PRL), compared to the control conditions (control + PRL) and treatment with the preA-derived CM (preA + PRL). The level of secreted alpha S1-casein in bMEC grown in CM collected from mature adipocytes (14 d + PRL) was lower than in preA + PRL, but higher than in control + PRL (the differences were not significant among these three experimental conditions). This suggests that the differentiated adipocytes may be more effective in regulating mammary epithelial cell secretory activity through paracrine actions. These observations also correspond with the results of the immunoenzymatic tests, analyzing the concentration of selected adipokines in the CM collected from adipocytes. The concentration of leptin was the highest in the CM derived from bovine adipocytes on day 14 of differentiation. It is possible that leptin was one of the active molecules that enhanced the alpha S1-casein secretion synergistically with PRL when bMEC were cultured in the 14 d + PRL CM. Previous studies by Feuermann et al. [43,62] showed that leptin enhanced the synthesis of alpha-casein and beta-lactoglobulin in the mammary gland explants from a lactating cow, but only in the presence of prolactin. It is important to note that adipocytes located in the stroma of the mammary gland can secrete more than 350 adipokines [63]; however, their exact function in paracrine regulation of mammary gland development and remodeling during the lactation cycle is yet to be established. Leptin, adiponectin, and VEGF are among the biologically active molecules whose expression is controlled by prolactin [21,64]. In our future studies, we are planning to analyze in more details the composition of the CM derived from preadipocytes and adipocytes to seek for active molecules that exert the strongest effect on bMEC physiology.

In conclusion, our in vitro study demonstrated that the paracrine interactions between adipocytes and bovine mammary epithelial cells had a positive impact on bMEC survival and stimulated the formation of larger spheroids by epithelial cells when cultured on reconstituted basement membrane (Matrigel). On the other hand, preadipocytes secreted paracrine factors that stimulated bMEC migration. The results confirm that preadipocytes and adipocytes are vital components of the stroma that forms the local microenvironment, secreting active molecules that regulate mammary parenchyma development in cattle. Identifying the specific stimulatory factors that play a key role in the paracrine interactions between bovine mammary epithelium and the surrounding stroma will require further research.

## 4. Materials and Methods

### 4.1. Tissue Sampling

Mammary tissue from udders and perirenal fat tissue from adipose capsule of kidneys were obtained at slaughterhouse from 4–6 year old non-pregnant Holstein–Friesian cows (Bos taurus) (*n* = 6), free of clinical signs of mastitis. All slaughterhouse animal procedures performed in this study were approved by county Veterinary Inspection. According to the Polish law, the post-mortem use of tissues does not require the approval from the Ethics Committee (the Act on the Protection of Animals Used for Scientific or Educational Purposes—Journal of Laws of Republic of Poland, abbreviated Dz.U.2015.266). The animals were not purchased for the purpose of this study and were not subjected to any experimental procedures. Tissue samples originated from a random selection of animals that arrived to the “Zdzieszyńska Danuta” slaughterhouse (Piekarska St 16, Wymysłów, Poland) at the day of samples collection. Tissue samples were collected within 20 min after slaughter and transported on ice to the cell culture laboratory of the Department of Physiological Sciences in Hank’s balanced salt solution (HBSS) with Ca2+ and Mg2+ (Sigma-Aldrich, part of Merck KGaA, Darmstadt, Germany), supplemented with antibiotics and antimycotics: penicillin-streptomycin (100 IU/mL), gentamycin (100 µg/mL), amphotericin B (5 µg/mL) (GibcoTM, Thermo Fisher Scientific, Waltham, MA, USA). Further procedures were performed under sterile conditions in laminar flow hood, intended for primary cell culture isolation.

### 4.2. Isolation of Bovine Mammary Epithelial Cells (bMEC)

Approximately 10 g of the mammary tissue was cut with a scalpel into 2–3 mm pieces, and then rinsed several times with fresh HBSS buffer with antibiotics in order to remove milk and blood remnants. Next, the mammary gland tissue pieces were placed in a digest mixture: HBSS with 0.5 mg/mL collagenase IV, 0.5 mg/mL hyaluronidase I-S, 0.4 mg/mL DNA-se I (all enzymes purchased from Sigma-Aldrich, Merck KGaA, Darmstadt, Germany), and antibiotics/antimycotics: penicillin-streptomycin (50 µg/mL), gentamycin (50 IU/mL), and amphotericin B (2.5 µg/mL) (Thermo Fisher Scientific, Waltham, MA, USA). After 1.5 h of incubation at 37 °C with constant shaking (150 rpm), cells were filtered through metal strainers (250 µm pore size, purchased from Sigma-Aldrich, Merck KGaA, Darmstadt, Germany) to remove larger tissue fragments, and were centrifuged for 5 min at 1500 rpm. Pellets were resuspended in HBSS solution with antibiotics/antimycotics, then filtered through cell strainers (70 µm pore size, BD Falcon, Franklin Lakes, NJ, USA), and centrifuged again for 5 min at 1500 rpm. Pellets were resuspended one more time in HBSS solution with antibiotics/antimycotics and filtered through cell strainers with smaller diameter (40 µm pore size, BD Falcon, USA) in order to obtain single cells suspension. After the last centrifugation (5 min at 1500 rpm), cell pellets were resuspended in growth medium (Dulbecco’s Modified Eagle Medium: Nutrient Mixture F-12: DMEM/F12) supplemented with 10% (*v*/*v*) heat-inactivated fetal bovine serum (FBS), insulin (1 µg/mL), hydrocortisone (1 µg/mL), holo-transferrin (5 µg/mL), and antibiotics/antimycotics (Sigma-Aldrich, Merck KGaA, Darmstadt, Germany). Resuspended cells were plated on sterile 75 cm^2^ tissue flasks (VWR International, Radnor, PA, USA) to allow cells to attach to the surface. Cell culture was conducted at 37 °C in tissue culture incubator with atmosphere of 5% CO_2_/95% humidified air, with the medium replaced every second day. All experiments were performed on cells from passage numbers: 3–6.

### 4.3. Real-Time RT-PCR—Primary Mammary Epithelial Cell Culture Evaluation

For RNA extraction, confluent bMEC were used at passages 3 and 4. Confluent cells were directly suspended in 400 μL of Fenozol Plus Buffer from the Total RNA mini-plus kit (A&A Biotechnology, Gdańsk, Poland) and stored at −80 °C until further use. Total RNA was extracted from cells with the Total RNA mini-plus kit (A&A Biotechnology, Gdańsk, Poland) according to the protocol provided by the producer. RNA concentration and purity were determined spectrophotometrically, and quality was confirmed using microcapillary electrophoresis (Bioanalyzer 2100, Agilent Technologies, Santa Clara, CA, USA). During reverse transcription, 2 μg of isolated total RNA was converted to cDNA with High Capacity cDNA Reverse Transcription Kit (Applied Biosystems, Thermo Fisher Scientific, Waltham, MA, USA), and the reaction was carried out in a Mastercycler pro (Eppendorf, Hamburg, Germany). Real-time PCR was performed in triplicate using SYBR Select Master Mix (Applied Biosystems, Thermo Fisher Scientific, Foster City, CA, USA). Each 10 μL reaction contained a final concentration of 0.5 μM of forward and reverse primers, 1x master mix, and 1 μL cDNA (100 ng). Reaction was performed in MX3005P QPCR Real-time PCR system (Stratagene, La Jolla, CA, USA). Cycling conditions started with two initial steps at 50 °C for 2 min and 95 °C for 2 min, followed by 40 cycles composed of denaturation (95 °C for 15 s), annealing (15 s at temperature dependent on the pair of primers designed), and extension phase (72 °C for 1 min). Standard curves were run for each transcript to ensure exponential amplification, and “no RT” controls were run to exclude nonspecific amplification. *RPS9* (ribosomal protein S9) was used as a reference gene. All primers: *KRT5, KRT14, KRT18, KRT19, RSP9* were purchased from PrimePCRTMSYBR^®^ Green Assay (Bio-Rad, Hercules, CA, USA). Detailed information about the commercial primers, including catalogue numbers are listed in Supplementary Table S1. The relative mRNA expression (delta Ct; ΔCt) of cytokeratin (*KRT*) genes was calculated by relating their respective Ct (cycle threshold) values to the mean Ct values of *RSP9* as follows: ΔCt = Ct target gene—Ct mean reference gene (*RSP9*). A lower ΔCt value corresponds to higher mRNA expression. The results were then expressed as (−ΔCt) + 10 for easier interpretation, as previously described by Ontsouka et al. [65]. The last step of calculations allows for obtaining results in which higher values correspond to higher mRNA expression.

### 4.4. Three-Dimensional Matrigel Cell Culture of bMEC

For 3D culture of bMEC, each Transwell insert (1 μm pore size) (BD Biosciences, San Jose, CA, USA) was coated with growth factor reduced Matrigel^TM^ (BD Biosciences, San Jose, CA, USA). Fifty microliters of Matrigel was spread on the surface of inserts’ porous membrane. Inserts covered with Matrigel were left to solidify for 30 min at 37 °C. Cells were plated at a concentration of 1 × 10^4^ cell/mL per single insert. Inserts were then placed in the 12-well companion plates, creating an upper chamber of each well. At the lower chamber, preadipocytes and adipocytes at different stages of differentiation (preA, d8, d12) were grown. A control culture of bMEC was created by placing the inserts with bMEC above wells that did not contain any cells at the lower chamber of the companion plates. On day 6 of mammospheres formation, series of micrographs from each insert were taken with the use of phase-contrast microscope: Leica DM IL LED (Leica, Wetzlar, Germany) to evaluate the growth rate and shape of bMEC spheroids. Series of measurements of mammospheres diameter were made using LAS X 3.3 Life Science Microscope Software (Leica, Wetzlar, Germany). The experiment was performed in three replicates. Each time, diameters of at least 10 mammospheres were measured for each experimental condition.

### 4.5. Isolation of Bovine Adipose-Derived Stem Cells (bASC) from Bovine Perirenal Fat Tissue

Approximately 10 g of perirenal tissue was obtained from adipose capsule of kidneys within 20 min after slaughter and transported to the cell culture laboratory at the same conditions and in the same transport buffer that was used for transportation of the bovine mammary tissue (described in Section 4.2). Fat tissue samples were sliced to 2–3 mm pieces, rinsed several times with fresh HBSS with antibiotics/antimycotics in order to remove blood remnants. Next, tissue pieces were placed in a digest mixture composed of HBSS enriched with collagenase type II (2 mg/mL), DNA-se I (0.4 mg/mL) (enzymes purchased from Merck—Sigma Aldrich), and antibiotics/antimycotics: penicillin-streptomycin (50 µg/mL), gentamycin (50 IU/mL), amphotericin B (2.5 µg/mL) (Thermo Fisher Scientific, Waltham, MA, USA). The digestion proceeded for 1–1.5 h in an orbital shaker (37 °C at 170 rpm). Cells were then filtered through cell strainers (70 µm pore size, BD Falcon, USA), and centrifuged again for 5 min at 800 rpm. Pellets contained stromal vascular cells that were resuspended in growth medium composed of DMEM/F12 medium supplemented with 10% (*v*/*v*) heat-inactivated new born calf serum (NBCS) (Thermo Fisher Scientific, Waltham, MA, USA) and antibiotics/antimycotics. Resuspended cells were then filtered through cell strainers with smaller diameter (40 µm pore size) in order to obtain single cells. After the last centrifugation (5 min at 800 rpm), pellets were resuspended in growth medium (DMEM/F12 supplemented with 10% (*v*/*v*) NBCS and antibiotics/antimycotics), plated on sterile 25 cm^2^ tissue flasks (VWR International, Radnor, PA, USA) and culture at 37 °C at conditions described in Section 4.2.

### 4.6. Adipogenic Differentiation

Adipogenic differentiation of bASC derived from the stromal vascular fraction isolated from the perirenal fat tissue was performed according to our own protocol established on the basis of available literature [66,67,68]. Cells were grown until 100% confluence in standard growth medium (DMEM/F12 supplemented with 10% (*v*/*v*) NBCS and antibiotics/antimycotics). Forty eight hours post-confluence, the adipogenic differentiation was induced by changing media to adipogenic medium I containing: DMEM/F12, 5% FBS, (50 µg/mL), antibiotics/antimycotics, and the following adipogenic factors: isobutylmethylxanthine (IBMX, 0.5 mM), dexamethasone (1 µM), rosiglitazone (2 µM)—purchased from Sigma-Aldrich (Merck KGaA, Darmstadt, Germany), and insulin-transferrin-selenium (ITS, 10 µM)—purchased from Thermo Fisher Scientific (Waltham, MA, USA). On day 3 of differentiation, the medium was replaced with adipogenic medium II containing DMEM/F12 supplemented with 5% (*v*/*v*) FBS, antibiotics/antimycotics, and two adipogenic factors: ITS (10 µM), rosiglitazone (2 µM). On day 6 of differentiation, the medium was replaced with the maintenance medium containing: DMEM/F12 with 10% (*v*/*v*) FBS and antibiotics, without any adipogenic factors. For the purpose of different analyses, cells and/or conditioned media (CM) were collected at indicated time points of adipogenic differentiation: day 0—undifferentiated preadipocytes (preA), day 8—poorly differentiated adipocytes (young adipocytes), day 12—well-differentiated adipocytes, day 14—mature adipocytes. In order to collect the conditioned media (CM), cells received fresh medium (DMEM/F12 with 10% NBCS and antibiotics for preadipocytes, DMEM/F12 with 10% FBS and antibiotics for adipocytes) 24 h before collection. The next day, CM were collected from the cells and centrifuged for 5 min at 800 rpm to remove the cellular debris. Next, supernatants were passed through syringe filters (pore size 0.22 µm) and stored at 4 °C before further use (for the period not longer than 2 weeks). CM media were never frozen and thawed to avoid degradation of the active compounds secreted to the medium.

### 4.7. Oil Red O Staining—Evaluation of Adipogenic Differentiation

Differentiated adipocytes (day 12 of differentiation) cultured in 6-well plates (BD Biosciences, San Jose, CA, USA) were washed with PBS and fixed with 3.7% paraformaldehyde (Sigma-Aldrich, Merck KGaA, Darmstadt, Germany) for 15 min at room temperature (RT). After rinsing in PBS, cells were incubated in 60% isopropanol (Avantor Performance Materials, Poland) for 5 min. Next, cells were washed in PBS and incubated for 20 min in 0.5% solution of Oil Red O (Sigma-Aldrich, Merck KGaA, Darmstadt, Germany) diluted in deionized water in a ratio 3:2. Next, cells were washed four times with water and visualized under a phase-contrast microscope Leica DM IL LED (Leica, Wetzlar, Germany).

### 4.8. Confocal Microscopy

Cells were plated on chamber slides (BD Biosciences, San Jose, CA, USA) at a concentration of 1 × 10^4^ cells/mL in standard culture medium. After a defined experimental period, cells were fixed with 3.7% paraformaldehyde (PFA) (Sigma-Aldrich, Merck KGaA, Darmstadt, Germany) for 15 min at RT and washed with PBS. Next, cells were permeabilized with 0.5% Triton X-100 diluted with PBS (Sigma-Aldrich, Merck KGaA, Darmstadt, Germany) for 10 min at RT, washed three times in PBS and incubated overnight with primary antibodies against MUC1 (cat. no: ab45167), cytokeratin 14 (cat. no: ab7800), or cytokeratin 19 (cat. no: ab52625) (Abcam, Cambridge, Cambridgeshire, the UK)—in the case of bMEC; CD 90 (cat. no: ab212885) or vimentin (cat. no: ab8978) (Abcam, Cambridge, Cambridgeshire, the UK)—in the case of preadipocytes. All antibodies were diluted in a ratio 1:100 with PBS. After overnight incubation with primary antibodies at 4 °C, cells were washed three times in PBS and incubated with Alexa Fluor 488-conjugated secondary antibodies (Invitrogen, Thermo Fisher Scientific, Waltham, MA, USA) diluted with PBS in a ratio 1:500 for 1 h in darkness at RT. Additionally, the cellular nuclei were stained with 7-aminoactinomycion D (7-AAD, 5 μg/mL) (Sigma-Aldrich, Merck KGaA, Darmstadt, Germany) and washed three times in PBS.

To evaluate the adipogenic differentiation of bASC, cells were fixed with 3.7% PFA, washed three times with PBS and incubated with fluorescent dye: HSC LipidTOX^TM^ Deep Red Neutral Lipid Stain (Invitrogen, Thermo Fisher Scientific, Waltham, MA, USA) diluted 1:200 with PBS. Afterwards, cells were washed three times in PBS and the cellular nuclei were stained with Hoechst 33342 (Sigma-Aldrich, Merck KGaA, Darmstadt, Germany). After each type of staining, the chambers were removed from the microscope slides, and coverslips were mounted on top of the slides using SlowFade^®^Gold mounting medium (Thermo Fisher Scientific, Waltham, MA, USA).

Cells were visualized using a confocal laser scanning microscope FV-500 system (Olympus Optical Co., Hamburg, Germany). The cells were examined using the Fluoview program (Olympus Optical Co., Germany). Five to ten pictures from each region of slide were analyzed. The analysis was performed in three replicates.

### 4.9. Immunoenzymatic Assays

Concentration of chosen adipokines was evaluated in conditioned media (CM) derived from preadipocytes (preA) and adipocytes at different stages of differentiation (8 d, 12 d, and 14 d) using commercially available immunoenzymatic tests purchased from MyBioSource, Inc. (San Diego, CA, USA): Retinoic acid receptor responder protein 2 ELISA kit—for chemerin detection (cat. no: MBS2885574), Bovine Adiponectin ELISA kit, (cat. no: MBS2881748), and Bovine Leptin ELISA kit (cat. no: MBS743525). Analyses were performed following the instructions provided by the producer. Concentration of alpha S1-casein was measured in cell lysates extracted from bMEC and in the media collected after bMEC culture. Prior to samples collection, bMEC were incubated with adipocytes-derived conditioned media (preA, 8 d, 12 d, and 14 d) for 24 h, with or without addition of prolactin (PRL, 3 µg/mL) (Sigma-Aldrich, Merck KGaA, Darmstadt, Germany). The next day, cells and media were collected. The media were centrifuged at 4 °C for 5 min at 800 rpm to remove the cellular debris. After centrifugation, supernatants were collected and stored at −80 °C until further analyses. Bovine mammary epithelial cells were removed from the culture flasks by scraping the cells in PBS. Next, samples were placed in centrifuge tubes, and centrifuged at 4 °C for 5 min at 800 rpm. Supernatants were removed, and cellular pellets were stored at −80 °C until further analyses. Protein extracts from cultured bMEC were isolated by lysing the collected cell pellets with RIPA buffer (50 mM Tris, pH 7.5, 150 mM NaCl, 1 mM EDTA, 1% NP-40, 0.25% Na-deoxycholate, and 1 mM PMSF) supplemented with protease inhibitor cocktail and phosphatase inhibitor cocktail (Sigma-Aldrich, Merck KGaA, Darmstadt, Germany) for 30 min at 4 °C. Lysates were cleared for 20 min at 14,000 rpm, and supernatants were collected. Protein concentration in the lysates was determined using Bio-Rad protein assay dye reagent according to the producer’s instructions (Bio-Rad, Hercules CA, USA). Alpha S1-casein concentration in protein extracts and collected media were analyzed with the use of immunoenzymatic test: Bovine Casein Alpha S1, ELISA kit (cat. no: MBS9358728) (MyBioSource, Inc., San Diego, CA, USA) according to the protocol provided by the producer. All samples were examined in triplicates and each experiment was conducted three times.

### 4.10. Cell Viability Assay

Cell viability was quantified by MTT assay. Bovine mammary epithelial cells were seeded onto 96-well plates (BD Biosciences, San Jose, CA, USA) at the concentration of 2 × 10^4^ cells per well. When cells reached 80–90% confluence, the medium was replaced with CM derived from preadipocytes (preA) or adipocytes at different stages of differentiation (8 d, 12 d, and 14 d). Cells were cultured in CM for 24 h. Next, bMEC were incubated with 0.5 mg/mL tetrazolium salt MTT diluted in phenol red-free DMEM/F12 medium (Sigma-Aldrich, Merck KGaA, Darmstadt, Germany) for 4 h at 37 °C. Afterwards, the medium was removed, cells were washed twice with PBS, and 100 μL of dimethyl sulfoxide (DMSO) (Sigma-Aldrich, Merck KGaA, Darmstadt, Germany) was added to each well to complete solubilization of the formazan crystals. Cell viability was quantified by measuring absorbance at 570 nm in a multi-well plate reader (Infinite 200 PRO TecanTM, TECAN, Männedorf, Switzerland). All samples were examined in triplicates and each experiment was conducted three times.

### 4.11. Cell Proliferation Assay

Proliferative activity of bMEC was quantified by CyQUANT^®^ Proliferation Assay Kit (Molecular Probes, Thermo Fisher Scientific, Waltham, MA, USA). bMEC were seeded onto 96-well microplates (BD Biosciences, San Jose, CA, USA) at the concentration of 1 × 10^4^ cells per well. When cells reached 50% confluence, the medium was replaced with CM derived from preadipocytes (preA) or adipocytes at different stages of differentiation (8 d, 12 d, and 14 d), and bMEC were cultured for subsequent 24 h. Then, media were removed, wells were washed with PBS, microplates with cells were frozen at −80 °C and stored until further analyses. On the day of analysis, microplates were thawed at room temperature and 200 μL of the dye/cell lysis buffer was added to each well according to the instructions of the producer. Next, samples were incubated for 2–5 min at room temperature, protected from light. Then, the fluorescence of each sample was measured using a microplate reader with filters appropriate for 480 nm excitation and 520 nm emission maxima (Infinite 200 PRO TecanTM, TECAN, Switzerland). All samples were examined in triplicates and each experiment was conducted three times (*n* = 9).

### 4.12. Annexin V Assay Analyzing the Number of Apoptotic Cell Death

Bovine mammary epithelial cells were seeded onto 6-well plates (BD Biosciences, San Jose, CA, USA) at the concentration of 1 × 10^5^ cells per well, and were grown until 70% confluence (BD Biosciences, San Jose, CA 95131, USA). Next, the medium was replaced with CM derived from preadipocytes (preA) or adipocytes at different stages of differentiation (8 d, 12 d, and 14 d), and bMEC were cultured for subsequent 24 h. In parallel, cells cultured in standard growth medium were used as control. Cells were trypsinized, centrifuged (at 4 °C for 3 min at 3000 rpm), and pellets were resuspended in 1 mL of PBS. Apoptosis was analyzed using Dead Cell Apoptosis Kit with Annexin V-Alexa Fluor^®^ 488 and propidium iodide (PI) (Molecular Probes, Thermo Fisher Scientific, Waltham, MA, USA) according to the protocol provided by the producer. Apoptotic cells, including those showing positive staining for Annexin V-Alexa Fluor^®^ 488 and negative for propidium iodide (PI) (early apoptosis) and those being double positive (late apoptosis) were counted using FACSAria II flow cytometer (BD Biosciences, USA) and presented as percentage of the total cell count. At least 2 × 10^4^ events were recorded per sample. The data were collected from four independent experiments.

### 4.13. Cell Migration Assay

Bovine mammary epithelial cells were seeded onto inserts with 8 μm pore membrane (BD FalconTM FluoroBlockTM 24-Multiwell Insert Plates) (Becton, Dickinson and Company, Franklin Lakes, NJ, USA) at the concentration of 5 × 10^4^ cells per well. Cells were grown in the upper chamber wells in the growth medium without FBS. The lower chamber wells were filled with conditioned media derived from preadipocytes (preA) or adipocytes at different stages of differentiation (8 d, 12 d, and 14 d), or three types of control medium: negative control without FBS, positive control with 10% (*v*/*v*) FBS, and positive control with 10% (*v*/*v*) NBCS. The cells were incubated in conditioned, or control media for 24 h. Next, the inserts were transferred into a second 24-well companion plate containing 500 μL/well of 2.5 μg/mL calcein AM (Sigma-Aldrich, Merck KGaA, Darmstadt, Germany) in HBSS, and incubated for 1 h at 37 °C. The fluorescence intensity of cells that passed through the membrane was analyzed at wavelengths of 490/515 nm (Ex/Em) on the bottom-reading fluorescent plate reader (Infinite 200 PRO TecanTM, TECAN, Switzerland). The background threshold for fluorescence intensity was set on a blank sample—insert without seeded cells, showing no fluorescence. Next, series of micrographs from each well were taken with the use of inverted fluorescence microscope (Olympus IX-71, Shinjuku, Tokyo, Japan). All samples were analyzed in triplicates and each experiment was conducted three times.

### 4.14. Statistical Analyses

Statistical analyses were performed using GraphPad PrismTM version 7.00 software (GraphPad Software, Inc., La Jolla, CA, USA). One-way analysis of variance (ANOVA) with Tukey’s multiple comparison post-test was used to determine the significance of effects between the treatments (control and different experimental conditions: preA, 8 d, 12 d, and 14 d) at a specific time point (24 h). In the case of data from the experiment determining the concentration of alpha S1-casein in bMEC treated with different conditioned media, with or without addition of PRL, the two-way analysis of variance (two-way ANOVA) with Tukey’s multiple comparison post-test was used to compare the effect of treatment in different experimental conditions (preA, 8 d, 12 d, and 14 d) and the effect of PRL. A *p* value of ≤0.05 was considered statistically significant, and *p* ≤ 0.01, *p* ≤ 0.001, or *p* ≤ 0.0001 as highly significant.

## Data Availability

The data presented in this study are available on request from the corresponding author. The data are not publicly available due to the continuity of the research on this topic.

## Supplementary Materials

The following supporting information can be downloaded at: https://www.mdpi.com/article/10.3390/ijms241713348/s1.
