# Supplementary material for: Adipocyte-Derived Paracrine Factors Regulate the In Vitro Development of Bovine Mammary Epithelial Cells
Source: Int J Mol Sci. 2023 Aug 28;24(17):13348. doi: 10.3390/ijms241713348 (PMC10487751; doi:10.3390/ijms241713348)
Supplement: Supplementary file 1 [file ijms-24-13348-s001.zip › Dziegelewska-Sokolowska et al._IJMS_2023_Table S1.pdf]

Table S1. : Information about PrimePCR™ SYBR® Green Assays (Bio-Rad, USA) used in real-time qPCR analyses.

Name of specific PrimePCR™ SYBR® Green Assay	Unique Assay ID
KRT5, Cow	qBtaCID0019936
KRT14, Cow	qBtaCID0004779
KRT18, Cow	qBtaCED0007607
KRT19, Cow	qBtaCID0004772
RPS9, Cow	qBtaCID0013542
