# Supplementary material for: Adipocyte-Derived Paracrine Factors Regulate the In Vitro Development of Bovine Mammary Epithelial Cells
Source: Int J Mol Sci. 2023 Aug 28;24(17):13348. doi: 10.3390/ijms241713348 (PMC10487751; doi:10.3390/ijms241713348)
Supplement: Supplementary file 1 [file ijms-24-13348-s001.zip › Dziegelewska-Sokolowska et al_IJMS_2023_Figure S1.pdf]

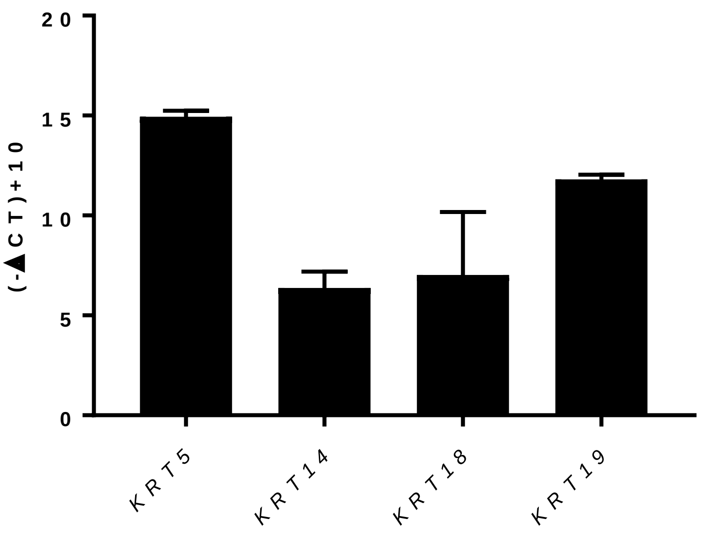

Figure S1. The expression of cytokeratin genes (*KRT5, KRT14, KRT18, KRT19*) in bMEC isolated from the bovine mammary gland. The relative mRNA expression of analyzed genes was normalized to the mean expression of *RPS9* reference gene. Data are shown as mean ± SD of the (−delta Ct)+10. The values are proportional to the gene expression levels. Bars indicate the standard deviation of two independent experiments measured in duplicates.
